# Cognitive and Neuroimaging Profiles of Older Adults With Attention Deficit/Hyperactivity Disorder Presenting to a Memory Clinic

**DOI:** 10.1177/10870547211060546

**Published:** 2021-11-16

**Authors:** Brandy L. Callahan, Nayani Ramakrishnan, Prathiba Shammi, Daniel Bierstone, Rebecca Taylor, Miracle Ozzoude, Maged Goubran, Donald T. Stuss, Sandra E. Black

**Affiliations:** 1 University of Calgary, Calgary, AB, Canada; 2 Hotchkiss Brain Institute, Calgary, AB, Canada; 3 University of Toronto, Toronto, ON, Canada; 4 Children’s Hospital of Eastern Ontario, Ottawa, ON, Canada

**Keywords:** neuropsychology, MRI, dementia, attention-deficit disorder, ADD, language

## Abstract

**Objective:**

Some features of attention-deficit/hyperactivity disorder (ADHD) may resemble those of mild cognitive impairment (MCI) in older adults, contributing to diagnostic uncertainty in individuals seeking assessment in memory clinics. We systematically compared cognition and brain structure in ADHD and MCI to clarify the extent of overlap and identify potential features unique to each.

**Method:**

Older adults from a Cognitive Neurology clinic (40 ADHD, 29 MCI, 37 controls) underwent neuropsychological assessment. A subsample (*n* = 80) underwent structural neuroimaging.

**Results:**

Memory was impaired in both patient groups, but reflected a storage deficit in MCI (supported by relatively smaller hippocampi) and an encoding deficit in ADHD (supported by frontal lobe thinning). Both groups displayed normal executive functioning. Semantic retrieval was uniquely impaired in MCI.

**Conclusion:**

Although ADHD has been proposed as a dementia risk factor or prodrome, we propose it is rather a pathophysiologically-unique phenotypic mimic acting via overlap in memory and executive performance.

## Introduction

Despite a 3% prevalence in later life (Michielsen et al., 2012), attention-deficit/hyperactivity disorder (ADHD) is not well-recognized in older adults (Adler et al., 2009). This may be because their subjective and objective cognitive difficulties are taken as signs of mild cognitive impairment (MCI) (Callahan et al., 2017; Ivanchak et al., 2012). Differential diagnosis of ADHD and MCI is perhaps particularly challenging in memory clinics where older adults seek assessment for concerns related to forgetfulness, absent-mindedness, difficulty multi-tasking or planning (i.e., concerns that characterize both adult ADHD [Kooij et al., 2019] and amnestic and non-amnestic MCI [Farias et al., 2006]). In principle, symptom onset should be the distinguishing feature between both disorders (Goodman, 2009), but there is evidence that most memory clinic practitioners do not systematically query about early-life behaviors that may indicate ADHD onset, and instead rely on cognitive testing (Fischer et al., 2012). This is problematic if there is no clear description of the cognitive profiles that characterize either disorder, or the extent of any overlap that may reduce diagnostic specificity.

Direct comparisons between ADHD and MCI are necessary to identify similarities (i.e., cognitive features of ADHD that are most likely to be taken for signs of MCI) and potential features unique to each disorder (i.e., that may be used to guide diagnostic impressions). Some authors have additionally suggested that structural neuroimaging may help distinguish ADHD from MCI (Goodman et al., 2016); however, for this recommendation to be empirically informed, it is necessary to expand our knowledge of structural neuroimaging profiles in ADHD aged >50 to determine which imaging features can be considered clinically useful. Complicating matters further are epidemiological reports linking ADHD symptoms to subsequent neurodegeneration (e.g., Golimstok et al., 2011), suggesting the distinction between ADHD and MCI may not be as clear-cut as one might assume if the former is a risk factor or prodromal stage of the latter. Characterizing the profiles of older adults with ADHD in relation to MCI is directly relevant to clinical practice, to inform diagnostic impressions and optimize the provision of clinical services to older adults.

Older adults with ADHD presenting to memory clinics may be a particularly relevant group to study. Although they are unlikely to be representative of community cases of ADHD, they may be a subgroup at higher risk for accelerated decline because they are seeking assessment for cognitive concerns. Subjective cognitive change—which is often the impetus for referral to a memory clinic (Blackburn et al., 2014)—has received increasing attention as a potential indicator of incipient neurodegeneration (Jessen et al., 2020). As such, studying older adults with ADHD presenting subjective complaints may provide relevant information regarding their cognitive and structural brain health with respect to potentially accelerated aging.

A second rationale for studying ADHD in a memory clinic is that, as described above, these individuals arguably comprise a group whose clinical presentation is the most challenging to distinguish from prodromal dementia, because their complaints and clinical presentation may be highly similar to MCI. Therefore, they represent a higher “threat” to diagnostic accuracy when attempting to distinguish degenerative from non-degenerative conditions (a key question in clinical practice). Community cases of ADHD, on the other hand, may be less likely to struggle with subjective or objective impairments in cognition, and may instead tend to present to mental health professionals for help with interpersonal problems, for example (Asherson et al., 2016). From this perspective, it may be somewhat less relevant to include them in a study of cognition. It is now well recognized that the clinical presentation of ADHD is extremely heterogeneous (Luo et al., 2019; Mostert et al., 2015; Wåhlstedt et al., 2009)—perhaps even more so in later life, after several decades of accumulated character-shaping individual experiences—and focusing scientific study on a relatively homogeneous group (i.e., those with cognitive complaints in later life) may yield more useful, interpretable findings.

For these reasons, this study aims to systematically compare the neuropsychological and neuroimaging characteristics of older adults with ADHD presenting to a cognitive neurology clinic, relative to clients with established MCI. We will first briefly summarize what is known about cognitive and structural brain features in each of these conditions.

The extent and severity of neuropsychological impairments in later-life ADHD remain unclear. Only three studies have included samples aged >50, and results were inconsistent: one reported deficits in frontal-lobe functions (Thorell et al., 2017), another found grossly normal cognition (Semeijn et al., 2015), and a case series reported fluctuating performance across 2 years (Klein et al., 2019). In younger adults, a recent review reported impairments in attention, episodic memory, and executive functions (LeRoy et al., 2019). Some studies have found no deficits whatsoever in younger adults (Bramham et al., 2012), or have found that low performance is primarily accounted for by depression (e.g., Semeijn et al., 2015). In MCI, cognitive deficits depend on the clinical subtype (i.e., single- vs. multi-domain amnestic or non-amnestic), but most commonly involve episodic memory, language, and executive functioning (Smith & Bondi, 2013).

Regarding neuroimaging in ADHD, to our knowledge, only one published study has involved older adults (*n* = 25) and reported reduced gray matter volume within the right medial frontal orbital area relative to age-matched controls (Klein et al., 2021). In younger adult samples, abnormalities primarily involve regions involved in modulating attention and executive processes, including volumetric and cortical thickness reductions in superior frontal, anterior cingulate, and orbitofrontal cortices (Paloyelis & Asherson, 2011). In MCI, structural brain abnormalities depend on the clinical subtype but may include atrophy within the lateral and medial temporal lobes, prefrontal and inferior parietal cortices, and posterior cingulate gyrus (Ghosh et al., 2014), and temporal and parietal cortical thinning (Bakkour et al., 2009).

These individual bodies of literature lead us to expect possible overlap between ADHD and MCI in memory and executive functions, as well as in prefrontal cortical volumes; this is the first study to compare these groups systematically.

## Methods

### Participants

**Figure 1.**
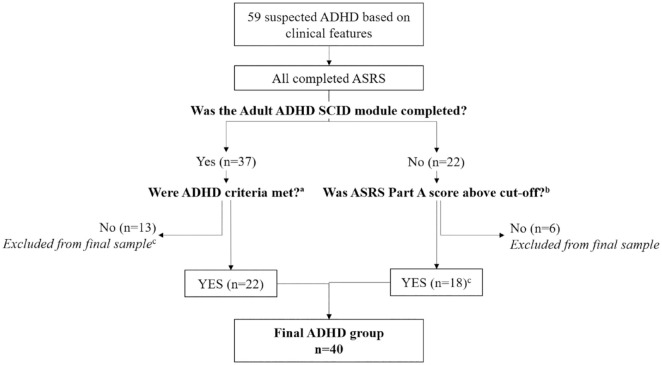
Flowchart illustrating ADHD classification.

Individuals were considered for inclusion if they were 50 to 85 years old. Fifty-nine participants suspected of ADHD were identified among patients presenting to a cognitive neurology clinic with cognitive concerns (Figure 1). All were screened using the Adult ADHD Self-Report Scale (ASRS, part A), and a subset (*n* = 37) also agreed to complete the ADHD module of the Structured Clinical Interview for DSM-5 (SCID-5). The remaining 22 were unavailable or refused to complete the SCID-5.

Participants were classified as “ADHD” if they met SCID-5 criteria for adult ADHD (*n* = 22). When SCID-5 had not been completed, ASRS scores were considered: 16 participants endorsed ≥4 items above a pre-specified threshold or obtained a total summed score of ≥14 and were considered to have significant ADHD symptoms (Kessler et al., 2007). All participants’ symptoms were ascertained as longstanding and corroborated using a “childhood symptoms” index, described below. Thirteen did not meet SCID-5 criteria, and six had no SCID-5 data available and screened negative on the ASRS; these 19 were excluded. Two reported longstanding attentional and executive issues and obtained ASRS ≥14; although they did not meet SCID-5 criteria because therapy had successfully helped them manage their symptoms’ functional impact, they were assumed to have underlying ADHD and included. Three ADHD participants were taking stimulant medication and 32 were not. Current medication was unavailable for 5.

Twenty-three MCI participants, who underwent testing as part of the Sunnybrook Dementia Study (SDS) (ClinicalTrials.gov NCT01800214), were selected as a comparison group. The SDS is a well-characterized cohort with neurodegenerative or vascular cognitive impairment. SDS participants are thoroughly screened to exclude secondary causes of impairment or concomitant illness; thus, any suspected cases of ADHD (i.e., with longstanding inattention, hyperactivity or impulsivity) were not included, and participants were stroke-free. Diagnoses are determined by at least two experienced clinicians based on neurological and cognitive examination. Data from an additional six MCI participants enrolled in another clinical study ongoing at Sunnybrook were also used here, bringing the total MCI sample to 29. All were diagnosed using Petersen’s (2004) criteria (subjective and objective impairment in any domain, preserved independence, no dementia). Based on Jak and Bondi’s (2009) comprehensive neuropsychological criteria, 3 had single-domain amnestic MCI, 14 had multiple-domain amnestic MCI, 2 had single-domain non-amnestic MCI, and 8 had multiple-domain non-amnestic MCI. Two had unclear profiles but evidenced isolated impairments in California Verbal Learning Test (CVLT) learning and recognition and Stroop color-naming, or Wisconsin Card Sorting Test (WCST) set-loss errors. Although cognitive tests (described below) were used to diagnose MCI and as primary outcome measures, their use in combination with ADHD screening measures (SCID or ASRS) aimed to decrease circularity (i.e., ADHD classification was made agnostic to cognitive status, allowing for the possibility that ADHD may be associated with clinically significant cognitive impairment [e.g., Golimstok et al., 2011]).

Data from an additional sample of 37 healthy controls, who also underwent testing in SDS, were used for comparison purposes. These participants reported no cognitive or behavioral complaints, performed within normal limits on all cognitive measures, and were stroke-free. The final sample consisted of 40 participants with ADHD, 29 with MCI and 37 controls.

### Measures of ADHD Symptoms

ADHD symptom measures were administered to all ADHD participants, and to a subset of 15 MCI participants who could be contacted following their SDS assessment. The remaining 15 MCI participants and 37 controls could not be reached to complete these measures at the time of this study.

Current symptom severity was assessed using the Conners Adult ADHD Rating Scale (CAARS) Self-Report Long Form. The ADHD Index, containing items that best distinguish ADHD from non-clinical cases (Conners et al., 1999), was the primary measure of interest. Raw scores were standardized to age-adjusted *T* scores using the technical manual (Conners et al., 1999).

Childhood symptoms were quantified using the Barkley Adult ADHD Rating Scale-IV (BAARS-IV) Self-Report Childhood Scale. This tool asks participants to rate the frequency of their inattentive and hyperactive/impulsive behaviors from age 5 to 12, providing two subscores which can then be summed to produce a “total ADHD” score. Raw scores were standardized to age-adjusted percentiles using the technical manual (Barkley, 2011a).

Participants with children were also queried about their children’s early-life ADHD symptoms using the BAARS-IV Other-Report Childhood Scale. This served as additional corroborative evidence of ADHD, as heritability studies estimate that ADHD occurs in 40% to 50% of parents who have a diagnosed child (Starck et al., 2016).

### Cognitive and Behavioral Measures

The Mini-Mental State Examination (MMSE) estimated global cognitive function. To quantify depressive symptoms, participants completed either the 30-item Geriatric Depression Scale (GDS) (*n* = 85), the Beck Depression Inventory II (BDI-II) (*n* = 14), or an informant completed the Cornell Scale for Depression in Dementia (*n* = 4). In four ADHD participants, the MMSE was completed >1 year after or prior to the rest of the neuropsychological assessment and was coded “missing.” One ADHD and two control participants did not complete any depression measure.

All participants underwent neuropsychological assessment, but because data were drawn from different sources not everyone completed all tests. Table 2 summarizes the number of participants having completed each test. Domains assessed included attention (forward digit span, Trails A, digit-symbol coding, Stroop word-reading, and color-naming), episodic memory (Logical Memory Short Story, CVLT, Rey-Osterrieth Complex Figure Task [ROCFT]), language (Boston Naming Test [BNT], phonemic and semantic fluency), and executive abilities (WCST, backward digit span, Stroop interference). Total time obtained on Trails B was transformed to a B/A ratio to isolate a relative measure of switching (Drane et al., 2002).

### Neuroimaging Measures

Twenty MCI and 33 controls had usable magnetic resonance neuroimaging (MRI) data collected withinSDS. Imaging data were acquired on 22 of the ADHD participants using the same protocol. Scans were acquired on a 1.5 Tesla GE Signa scanner (Milwaukee, WI, USA) and included a T1-weighted axial three-dimensional spoiled gradient recalled echo (5 ms echo time [TE], 35 ms repetition time [TR], 1 number of excitations [NEX], 35° flip angle [FOV], 22 × 16.5 cm, 0.859 × 0.859 mm in-plane resolution, with 1.2–1.4 mm slice thickness depending on head size) and interleaved PD and T2 sequences (interleaved axial dual-echo spin echo: TEs of 30 and 80 ms, 3 s TR, 0.5 NEX, 20 × 20 cm FOV, 0.781 × 0.781 mm in-plane resolution, 3 mm slice thickness).

Cortical thickness analysis (Fischl & Dale, 2000) was conducted using an enhanced modification of FreeSurfer software v.6.0 (http://surfer.nmr.mgh.harvard.edu/). Pre-processing of T1-weighted scans included motion correction, skull-stripping, transformation to Talairach space, intensity normalization, hemispheric separation, and tissue segmentation and parcellation. Two additional stages improved segmentation accuracy and quality assessment based on the PD/T2 sequence (Ozzoude et al., 2020): Stage 1 involved replacing the skull-stripped brain in FreeSurfer with one generated using a semi-automatic brain extraction (SABRE) pipeline (Dade et al., 2004) which enhances the overall downstream processes in FreeSurfer. Stage 2 involved incorporating lesion masks from a PD and T2-based lesion segmentation pipeline to account for significant atrophy and small vessel disease (Ramirez et al., 2011). Gray (GM) and white matter (WM), and GM and cerebrospinal fluid (CSF) borders, were identified and modeled as surfaces. Cortical thickness was defined as the distance between the GM and WM surface boundaries and the GM and CSF boundaries along each point of the cortex in each hemisphere. After pre-processing, surface data were resampled to FreeSurfer’s average surface map. A 15-mm full-width half-maximum Gaussian spatial smoothing kernel was applied to the surface maps. FreeSurfer outputs were visually quality-controlled based on the Desikan et al. (2006) atlas parcellation.

T1 was segmented using a multi-feature histogram method to generate a tissue segmentation containing normal appearing GM (NAGM), normal appearing WM (NAWM), sulcal and ventricular CSF (Kovacevic et al., 2002). SABRE was used to parcellate brain tissue into 26 standardized volumes of interest described elsewhere (Dade et al., 2004). Hippocampal volumes were segmented using a 3D convolutional neural network with a U-net architecture that is robust for populations with brain atrophy (https://hippmapp3r.readthedocs.io) (Goubran et al., 2020).

### Statistical Analyses

Depressive symptoms were categorized as “none” (GDS < 10; BDI-II < 14; Cornell < 8), “mild/probable” (GDS 10–19; BDI-II 14–19; Cornell 8–12) or “moderate/severe” (GDS > 19; BDI-II > 19; Cornell > 12). The groups were compared using Kruskal-Wallis H (age, education, MMSE), chi-square (sex, depression), or *t*-tests (ADHD symptom scores).

Raw cognitive scores were standardized to *Z*-scores using published normative data, and NAGM volumes in inferior, middle, and superior frontal regions, superior and inferior parietal regions, and anterior and posterior temporal regions were corrected for head size (regional mm^3^/total supratentorial intracranial volume). Transformed scores were entered into separate univariate ANOVAs adjusted for age, sex, MMSE and depressive symptoms. Non-significant predictors were removed except age, and pairwise comparisons were examined where main effects were significant.

For cortical thickness analyses, vertex-wise surface-based analysis was first performed within the frontal lobe only using the general linear model in FreeSurfer, based on cortical thickness alterations in young adults with ADHD. A second exploratory analysis was performed across the whole brain. Age, education, and MMSE scores were included as regressors of no interest. Monte Carlo simulation with 5000 iterations using a cluster-wise probability (*p*_(cwp)_) of *p* < .05 (two-sided) was used to correct for multiple comparisons. Bonferroni correction was applied across the two hemispheres.

## Results

### Participant Characteristics

The groups differed on age, sex, MMSE, and depressive symptoms, but not education (Table 1). By design, participants with ADHD obtained higher current and childhood symptom scores. Higher children’s BAARS scores corroborate our ADHD classification.

**Table 1.** Participant Characteristics.

	Controls		ADHD		MCI		Test statistic	*p*-Value	Effect size
	*N*	Mean (*SD*)	*N*	Mean (*SD*)	*N*	Mean (*SD*)			
Age (years)	37	67.3 (7.1)	40	64.0 (8.9)	29	73.7 (6.5)	χ^2^ = 22.212	<.001	φ = .458
Sex (% women)	37	78.4%	40	52.5%	29	72.4%	χ^2^ = 6.353	.042	φ = .245
Education (years)	37	15.9 (2.4)	40	16.2 (3.7)	29	15.2 (3.1)	χ^2^ = 2.030	.362	φ = .138
Depressive symptoms	35		39		29		χ^2^ = 14.666	.005	φ = .377
% None (GDS < < span>10; BDI-II < < span>14)		91.9%		62.5%		82.8%			
% Mild/probable (GDS 10–19; BDI-II 14–19)		2.7%		30.0%		10.3%			
% Moderate/severe (GDS > 19; BDI-II > 19)		0.0%		5.0%		6.9%			
MMSE (total score)	37	29.1 (0.9)	36	28.2 (1.6)	29	27.2 (1.8)	χ^2^ = 22.020	<.001	φ = .465
ASRS (total score, part A)		N/A	40	15.3 (4.2)	14	7.1 (3.5)	*t =* 6.483	<.001	*d* = 2.070
CAARS (ADHD index T-score)		N/A	39	57.1 (10.1)	15	46.3 (7.4)	*t =* 3.724	<.001	*d* = 1.165
BAARS (score for highest-scoring child)		N/A	30	34.4 (12.3)	10	23.0 (6.1)	*t =* 3.880	<.001	*d* = 1.049

*Notes.* Effect sizes refer to phi (φ) where .1, .3, and .5 indicate small, medium, and large effect sizes, and to Cohen’s *d* where 0.2, 0.5 and 0.8 indicate small, medium, and large effect sizes. Because data were drawn from different sources, not all of the participants completed the ASRS, CAARS, and BAARS; the *N* columns summarize the number of participants having completed each questionnaire. The ASRS has a maximum score of 24, where higher values indicate more severe ADHD symptoms and scores ≥14 are generally indicative of clinically significant ADHD symptoms. CAARS values have been transformed to T-scores, where scores ≥65 are generally indicative of clinically significant ADHD symptoms. Bold text indicates statistical significance. ADHD = attention-deficit/hyperactivity disorder; ASRS = Adult ADHD Self-Report Scale; BAARS = Barkley Adult ADHD Rating Scale IV; CAARS = Conners Adult ADHD Rating Scale Self-Report Long Form; MCI = mild cognitive impairment; MMSE = mini mental state examination; *SD* = standard deviation.

### Cognitive Measures

Relative to controls, MCI participants performed worse on measures of attention (digit-symbol coding and Stroop color-naming), all memory measures (except immediate Short Story recall and CVLT recognition), language (BNT and semantic fluency), and one measure of executive functioning (Trails switching). CVLT free recall and ROCFT recall were also worse in MCI than in ADHD. ADHD participants were impaired on digit-symbol coding, Stroop color-naming, and immediate and delayed recall of the Short Story. Performance on all other tasks was comparable between groups (Table 2).

**Table 2.** Cognitive Performance.

	Controls		ADHD		MCI		*F* value	*p*-Value	Partial eta^2^
	*N*	Mean (*SD*)	*N*	Mean (*SD*)	*N*	Mean (*SD*)			
Attention and processing speed									
Forward span	37	0.08 (0.87)	40	−0.41 (1.10)	29	−0.36 (0.86)	1.163	.317	.023
Trails A time	37	−0.18 (0.66)	40	−0.66 (1.27)	29	−0.38 (0.98)	1.921	.152	.036
Coding	37	0.62 (0.92)	27	−0.33 (0.87)	22	0.08 (0.91)	9.661^a,b^	<**.001**	.191
Stroop color naming time	36	0.14 (0.74)	25	−0.69 (1.07)	26	−0.65 (0.85)	9.417^a,b^	<**.001**	.185
Stroop word reading time	36	−0.12 (0.75)	25	−0.68 (1.01)	26	−0.70 (0.76)	1.941	.150	.046
Episodic memory									
Logical memory immediate recall	35	0.39 (0.74)	26	−0.59 (1.24)	20	−0.52 (0.99)	4.439^b^	**.015**	.110
Logical memory delayed recall	35	0.51 (0.77)	26	−0.57 (1.16)	20	−0.70 (1.20)	5.792^a,b^	**.005**	.139
CVLT short delay free recall	36	0.33 (0.83)	29	−0.43 (1.42)	23	−1.52 (1.30)	7.559^a,c^	**.001**	.161
CVLT short delay cued recall	36	0.33 (0.68)	29	−0.67 (1.50)	23	−1.28 (1.12)	6.240^a^	**.003**	.136
CVLT long delay free recall	36	0.36 (0.80)	29	−0.40 (1.31)	23	−1.74 (1.29)	16.781^a,c^	<**.001**	.309
CVLT long delay cued recall	36	0.17 (0.77)	29	−0.53 (1.51)	23	−1.46 (1.24)	5.006^a^	**.009**	.112
CVLT recognition hits	36	0.28 (0.74)	29	−0.60 (1.23)	23	−0.48 (0.91)	2.595	.081	.062
CVLT recognition false positives	36	−0.25 (0.94)	29	0.38 (1.32)	23	1.07 (1.64)	2.583	.082	.062
ROCFT immediate recall	37	0.92 (1.27)	26	0.34 (1.45)	22	−0.41 (1.21)	9.346^a,c^	<**.001**	.188
ROCFT delayed recall	37	0.79 (1.29)	26	0.16 (1.47)	21	−0.73 (1.42)	5.469^a,c^	**.006**	.127
Language									
BNT	37	−0.09 (0.55)	29	−0.39 (0.84)	23	−1.13 (1.69)	6.214^a^	**.003**	.128
Semantic fluency	37	−0.10 (0.81)	38	−0.53 (0.97)	29	−1.15 (0.72)	10.304^a^	<**.001**	.171
Phonemic fluency	37	0.79 (0.75)	31	0.37 (1.12)	29	0.28 (0.96)	0.537	.587	.012
Executive functions									
Backward span	37	0.12 (1.14)	40	−0.14 (0.95)	29	0.01 (0.80)	0.393	.676	.008
Trails B/A ratio	37	−0.78 (0.66)	40	−0.05 (1.68)	28	0.09 (1.01)	5.340^a^	**.006**	.096
WCST category completion	37	−1.08 (0.22)	40	−1.19 (0.33)	29	−1.08 (0.23)	0.866	.424	.017
WCST set loss errors	37	−1.14 (0.42)	39	−1.10 (0.32)	29	−1.29 (0.54)	1.173	.314	.024
Stroop interference time	36	0.51 (0.78)	25	−0.24 (1.00)	26	−0.10 (1.04)	2.077	.132	.049

*Notes.* Because data were drawn from different sources, not all participants completed the full battery of cognitive measures; the *N* column summarizes the number of participants having completed each test. Bolded values highlight statistically significant main effects of Group. ADHD = attention-deficit/hyperactivity disorder; BNT = Boston naming test; CVLT = California verbal learning test; MCI = mild cognitive impairment; ROCFT = Rey-Osterrieth complex figure test; *SD* = standard deviation; WCST = Wisconsin card sorting test.

a MCI different from controls.

b ADHD different from controls.

c ADHD different from MCI.

### Neuroimaging Measures

**Figure 2.**
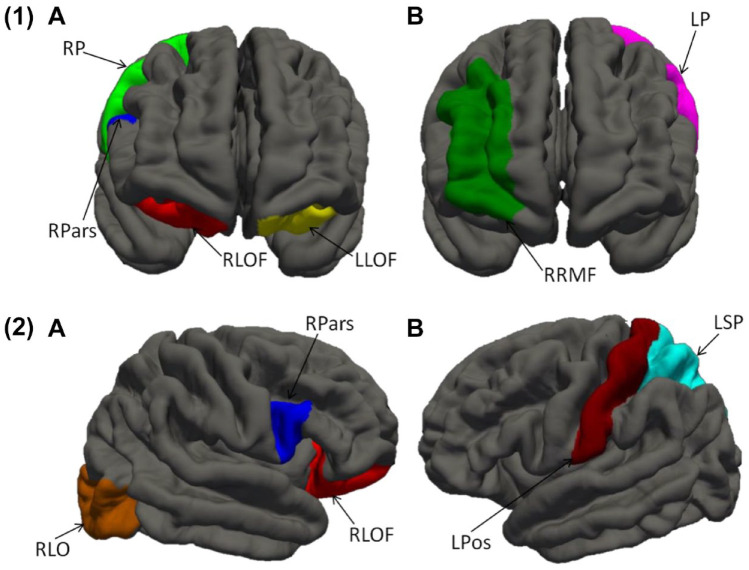
Frontal-lobe (1) and whole-brain (2) cortical thickness analysis showing frontal regions with decreased cortical thickness in (A) ADHD relative to controls and (B) ADHD relative to MCI. *Notes*. ADHD = attention-deficit/hyperactivity disorder; LLOF = left lateral orbitofrontal; LP = left precentral; LPos = left postcentral; LSP = left superior parietal; MCI = mild cognitive impairment; RLO = right lateral occipital; RLOF = right lateral orbitofrontal; RP = right precentral; RPars = right pars opercularis; RRMF = right rostral middle frontal.

The groups’ mean NAGM volumes did not differ in any region of interest, except hippocampal volumes which were marginally lower in MCI participants than controls (Table 3). In frontal-lobe cortical thickness analyses (Table 4, Figure 2(1)), ADHD participants showed decreased thickness in right precentral, right pars opercularis, and bilateral lateral orbitofrontal cortices compared to controls. They also showed reduced thickness in the right rostral middle frontal and left precentral cortices relative to MCI. In whole-brain analyses (Table 4, Figure 2(2)), cortical thickness in right lateral orbitofrontal, right lateral occipital, and right pars opercularis cortices was reduced in ADHD relative to controls. ADHD participants also had lower cortical thickness in the left postcentral and left superior parietal cortices compared to MCI.

**Table 3.** Regional Brain Volumes (in mm^3^), Corrected for Total Intracranial Volume.

	Controls		ADHD		MCI		*F* value	*p-*Value	Partial eta^2^
	*N*	Mean (*SD*)	*N*	Mean (*SD*)	*N*	Mean (*SD*)			
Total NAGM	33	0.464 (0.022)	22	0.458 (0.023)	20	0.447 (0.025)	2.768	.070	.073
Superior frontal NAGM	33	0.024 (0.002)	22	0.023 (0.003)	20	0.023 (0.003)	1.766	.178	.047
Anterior cingulate NAGM	33	0.024 (0.002)	22	0.024 (0.002)	20	0.023 (0.003)	0.554	.577	.016
Inferior frontal NAGM	33	0.030 (0.005)	22	0.030 (0.006)	20	0.028 (0.005)	0.546	.582	.015
Inferior parietal NAGM	33	0.059 (0.009)	22	0.057 (0.010)	20	0.060 (0.007)	0.823	.443	.023
Posterior cingulate NAGM	33	0.049 (0.006)	22	0.050 (0.008)	20	0.046 (0.007)	2.078	.133	.056
Anterior temporal NAGM	33	0.026 (0.003)	22	0.025 (0.002)	20	0.025 (0.004)	0.973	.383	.027
Posterior temporal NAGM	33	0.099 (0.008)	22	0.099 (0.008)	20	0.094 (0.006)	0.788	.459	.022
Hippocampal volumes	31	0.006 (0.001)	11	0.005 (0.001)	14	0.005 (0.001)	3.318	.044^a^	.115

*Notes.* Effect sizes refer to partial eta squared (η^2^_p_), where .01, .06, and .14 indicate small, medium, and large effect sizes. All volumes are reported in mm^3^ and have been corrected for total intracranial volume. Because data were drawn from different sources, not all participants completed neuroimaging; the *N* column summarizes the number of participants with available imaging data. Bolded values highlight statistically significant main effects of group. ADHD = attention-deficit/hyperactivity disorder; MCI = mild cognitive impairment; NAGM = normal-appearing gray matter; *SD* = standard deviation.

a MCI marginally different from Controls (*p* = .053).

**Table 4.** Whole Brain and Frontal Lobe Analyses Showing Significant Clusters With Cortical Thinning Corrected for Multiple Comparisons.

	Anatomical regions	Max-log10 (*p*-value*)*	Surface area of cluster (mm^2^)	Talairach (MNI305) coordinates (*x*, *y*, *z*)	LowCWP–HiCWP	*p*_(cwp)_
Frontal lobe analyses						
ADHD< NC	Right precentral	3.164	594.62	47.2, 0.4, 32.9	0.003–0.007	.005
	Right pars opercularis	3.189	756.69	38.9, 17.8, 20.5	0.000–0.001	<.001
	Right lateral orbitofrontal	3.928	1147.90	27.2, 34.3, −7.8	0.000–0.001	<.001
	Left lateral orbitofrontal	4.866	561.65	−28.7, 28.2, −16.0	0.009–0.015	.012
ADHD< MCI	Right rostral middle frontal	−3.751	617.16	42.4, 21.5, 32.6	0.003–0.007	.005
	Left precentral	−3.137	761.69	−35.9, −16.3, 53.7	0.000–0.016	<.001
Whole brain analyses						
ADHD < NC	Right lateral orbitofrontal	3.927	1149.61	27.2, 34.3, −7.8	0.002–0.005	.003
	Right lateral occipital	4.461	2482.53	43.8, −73.8, −7.7	0.000–0.001	<.001
	Right pars opercularis	3.189	756.69	38.9, 17.8, 20.5	0.035–0.045	.039
ADHD < MCI	Left postcentral	−3.566	1023.82	−51.0, −17.9, 52.9	0.006–0.010	.008
	Left superior parietal	−3.873	1003.53	−26.9, −60.5, 45.2	0.008–0.012	.009

*Notes.* Bolded values highlight statistically significant main effects of group. LowCWP = Lower clusterwise *p*-value 90% confidence interval; HiCWP = upper clusterwise *p*-value 90% confidence; MCI = mild cognitive impairment; NC = normal control; ADHD=Attention-deficit/hyperactivity disorder; *p*_(cwp)_ = clusterwise *p*-value.

## Discussion

This study aimed to quantify the shared and unique cognitive and imaging characteristics of older adults with ADHD or MCI presenting to a memory clinic. First, we found notable overlap in participants’ recall of a short story. However, ADHD participants’ performance was statistically comparable to controls’ in a context with added semantic structure (the CVLT), suggesting a frontally-mediated encoding deficit in ADHD and a temporally-mediated storage deficit in MCI. This interpretation is corroborated by compromised frontal and medial temporal lobe brain structures in our samples of ADHD and MCI, respectively, on neuroimaging.

Only MCI participants showed robust semantic retrieval deficits (naming and semantic fluency) relative to controls. Semantic abilities have been normal in previous investigations of later-life ADHD (Semeijn et al., 2015) and in some younger adult cohorts (Bueno et al., 2017). These rely relatively more on anterior temporal than frontal brain regions (Jackson et al., 2016) and may thus be most sensitive to the earliest stages of dementia due to Alzheimer’s disease (AD), which is known to begin in temporal regions (Braak & Braak, 1995).

Both ADHD and MCI groups showed grossly normal executive performance relative to controls. Similarly, three previous studies of ADHD in individuals aged >50 found normal executive performance when considered on a standardized scale (i.e., scaled scores between 8.3 and 10.9) (Thorell et al., 2017) and relative to controls (Semeijn et al., 2015), or normalization of initial deficits over time (Klein et al., 2019). Despite normal test scores, our ADHD group did endorse symptom items closely tied to executive functioning on the CAARS (e.g., wrapping up project details, organizing tasks). This corroborates previous observations that “executive” occupational impairments in ADHD are not adequately captured by formal testing (Barkley & Murphy, 2010). Fractionation of frontal-lobe functions in future work may be relatively more informative in ADHD/MCI comparisons, because our findings and others’ (Ghosh et al., 2014; Paloyelis & Asherson, 2011) suggest that frontal regions associated with process-specific specialization are differentially affected in both conditions. It will also be worthwhile for future work to examine the functional impact of executive failures, for example using the Barkley Deficits in Executive Functioning (BDEFS; Barkley, 2011b), which quantifies the extent of daily difficulties (e.g., with time management and everyday organization) and may be a more meaningful measure of executive difficulties than formal neuropsychological measures (Barkley, 2019). Further, there is recent evidence that functional impairment associated with ADHD may fluctuate within a single individual over time (Sibley et al., 2021), and this may be particularly relevant to examine in older adults who may have developed adaptive functional strategies that may have been more or less effective at different time points and in different contexts throughout their life.

Hippocampal volumes were relatively reduced in MCI, tentatively supporting an interpretation of a primary (storage) memory deficit. The ADHD group showed cortical thinning in the middle frontal gyrus which mediates working memory abilities (Petrides et al., 1993) and sustained mnemonic responses (Leung et al., 2002). Cortical thinning in the pars opercularis is rather more difficult to interpret, but may be understood in the context of reduced verbal memory retrieval in our ADHD cohort, as right inferior frontal gyrus (which comprises the pars opercularis) has been linked to verbal learning and recall (Sowell et al., 2007). Alternatively, cortical thinning of the right pars opercularis has been associated with poor motor inhibition (Curley et al., 2018) which, although we did not measure it in this study, is often impaired in adult ADHD (Carr et al., 2006). The lateral orbitofrontal cortex, which was also thinner in our ADHD sample relative to controls, has been implicated in decision-making and judgment (Nogueira et al., 2017), which were not explicitly assessed in the present study.

### ADHD: Phenotypic Mimic of MCI?

Previous studies have reported associations between ADHD symptoms and neurodegeneration in later life (e.g., Golimstok et al., 2011), leading some authors to speculate that ADHD may be a risk factor for dementia, or represent an early point along a neurodegenerative pathophysiological continuum, potentially characterized by hypodopaminergic brain states (Golimstok et al., 2011). Our data indicate that, indeed, both disorders display broadly overlapping dementia-like features (i.e., impaired aspects of verbal episodic memory). Careful examination, however, can tease apart unique characteristics (i.e., separate storage and encoding impairments in MCI and ADHD, respectively). Considered alongside additional MCI-specific semantic weaknesses and unique neuroimaging markers (i.e., frontal-lobe thinning in ADHD and marginally smaller hippocampi in MCI), these findings lead us to hypothesize that the pathological processes underlying ADHD and MCI are fundamentally distinct, and that their putative association in prior work is more parsimoniously explained by ADHD mimicking the MCI phenotype (Callahan et al., 2017). Attentional processes play a central role in successful memory functioning through their role in facilitating encoding (Aly & Turk-Browne, 2017), and deficits in some of these processes appear to manifest as memory impairment in ADHD. Because memory deficits are considered the hallmark feature of MCI (particularly MCI due to AD), they may lead older adults with ADHD to seek assessment or treatment in memory clinics (Fischer et al., 2012) and contribute to diagnostic confusion with MCI/early dementia (Goodman et al., 2016).

It follows from this hypothesis that, in addition to unique cognitive and neuroimaging profiles, ADHD and MCI should show specific physiological and pathological markers of disease, and distinct longitudinal cognitive trajectories. Some important next tests of this hypothesis, then, may include quantification of various neurodegenerative biomarkers (e.g., using functional and diffusion MRI data, molecular imaging, or emerging plasma biomarkers) and prospective follow-up of participants with ADHD to determine the extent to which their cognitive and structural trajectories resemble those of normal versus abnormal aging.

In this study, the possibility cannot be ruled out that ADHD and MCI may indeed be different points along a single neurodegenerative continuum as others have proposed (Golimstok et al., 2011) and that the between-group cognitive and structural differences seen here may be explained by differences in disease severity. We posit this is unlikely, because our neuroimaging results point to unique cortical thinning patterns only present in ADHD (that one would expect to see in MCI if it were a “later” point on the same disease continuum), though this could be because we enrolled primarily participants with MCI due to AD. For example, early Parkinson’s disease is associated with frontal-lobe thinning patterns similar to those observed in our ADHD group (Wilson et al., 2019). Thus, the hypothesis we propose should be tested in replication samples as well as other neurodegenerative disorders besides AD.

### Limitations

An important limitation of our sampling method is that many MCI and control participants could not be reached to complete ADHD symptom measures. Nonetheless, we estimate that undetected ADHD in either comparison sample is unlikely, as all participans underwent comprehensive assessment by at least two experienced clinicians, during which MCI participants were determined to have experienced a *recent* onset of cognitive change, and healthy controls were determined to be free of any current or chronic cognitive or behavioral concerns. Because *chronic* inattention/impulsivity are core features of ADHD (American Psychological Association, 2013) and were found absent by both expert clinicians, we have reasonable confidence that no ADHD cases went undetected in these groups.

Neuroimaging was only available on a small subsample, and we did not collect functional or diffusion MRI data. We therefore interpret these findings cautiously as a discovery sample requiring replication that will stimulate further research on this relatively common condition that can complicate brain aging and masquerade as neurodegeneration and may benefit from cognitive rehabilitation.

## Conclusion

Memory is impaired in both later-life ADHD and MCI, but frontal-lobe cortical thinning in ADHD versus hippocampal atrophy in MCI support process-specific impairments contributing to memory deficits. In contrast to previous reports of ADHD as possible risk factor or prodrome of dementia, we propose the hypothesis that this association may be more parsimoniously explained by ADHD “mimicking” the MCI phenotype via distinct pathophysiological processes. Prospective follow-up of ADHD participants, particularly relative to non-AD groups, are necessary to ascertain whether ADHD is associated with accelerated cognitive decline (e.g., via α-synucleinopathy or vascular burden accumulation).
